# Endoscopic virtual ruler based on artificial intelligence technology

**DOI:** 10.1055/a-2598-4576

**Published:** 2025-06-13

**Authors:** Yaxian Kuai, Zhihong Wang, Xuecan Mei, Zhuang Zeng, Yuchuan Bai, Aijiu Wu, Derun Kong

**Affiliations:** 1 36639 Key Laboratory of Digestive Diseases of Anhui Province, Department of Gastroenterology, The First Affiliated Hospital of Anhui Medical University, Hefei, China; 2 Research and Development Department, Hefei Zhongna Medical Instrument Co. Ltd., Hefei, China

Measurement and evaluation of lesion size has always been one of the difficult problems to solve in endoscopy 1 . Recently, we developed a real-time endoscopic virtual ruler measurement technology based on artificial intelligence (AI) technology.

Geometric optics showed that the distance of the object was the only variable affecting the ratio of the size of the object to the size of the image when the focal length was fixed and the lesion was centered (the transverse distance from the main optical axis was zero) 2 . Therefore, to obtain the actual size of the lesion, it is only necessary to measure the distance from the end of the lens to the lesion (object distance) and calculate the ratio of the size of the object to that of the image ( Fig. 1 ). This detection method can now be implemented using the image recognition technology of computer vision.

**Fig. 1:**
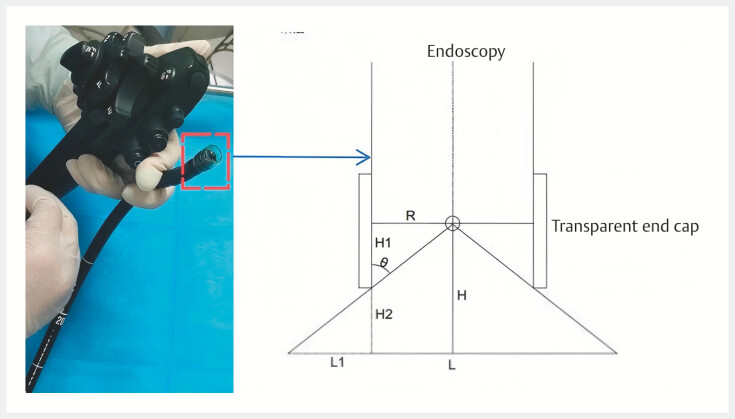
Schematic diagram of the endoscopic virtual ruler. The radius of the endoscope is *R* , the height of the transparent cap above the endoscopy is *H*_1_ , the diameter of the measured object is *L* , and the increased distance between the tip of the endoscope and the measured object is *H*_2_ (this distance can be obtained from the scale on the endoscope), *L* = *R* + *H*_1_ ×( *H* − *H*_1_ )/ *R* .

To do this, first install the transparent cap at the front end of the endoscope (10 mm/13 mm internal diameter) and adjust the distance of the cap end to the lesion until the lesion is fully within the field of view of the transparent endoscope cap, as the size of the inner diameter of the transparent cap is known. At this point, simply enter the distance from the front end of the endoscope to the maximum diameter of the lesion in the measurement system (recorded by an endoscope scale), and the measurement system will analyze the image of the target, forming a ruler that can measure the size of the lesion ( Fig. 2 ).

**Fig. 2:**
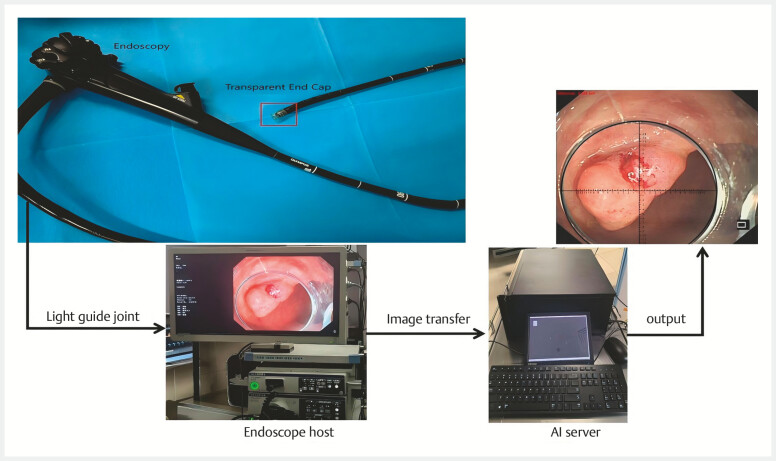
Schematic diagram of the operation process of the endoscopic virtual ruler.

Various clinical cases were studied ( Video 1 ) after comparing the scale of the virtual ruler with that of the physical ruler at different distances in vitro ( Fig. 3 ). The size of polyps and early cancer lesions could be well evaluated by using the model. Doctors can use virtual ruler measurement technology to measure lesions effectively during endoscopy.

The endoscopic virtual ruler is introduced and its clinical application shown for various types of lesions and at different distances.Video 1

**Fig. 3:**
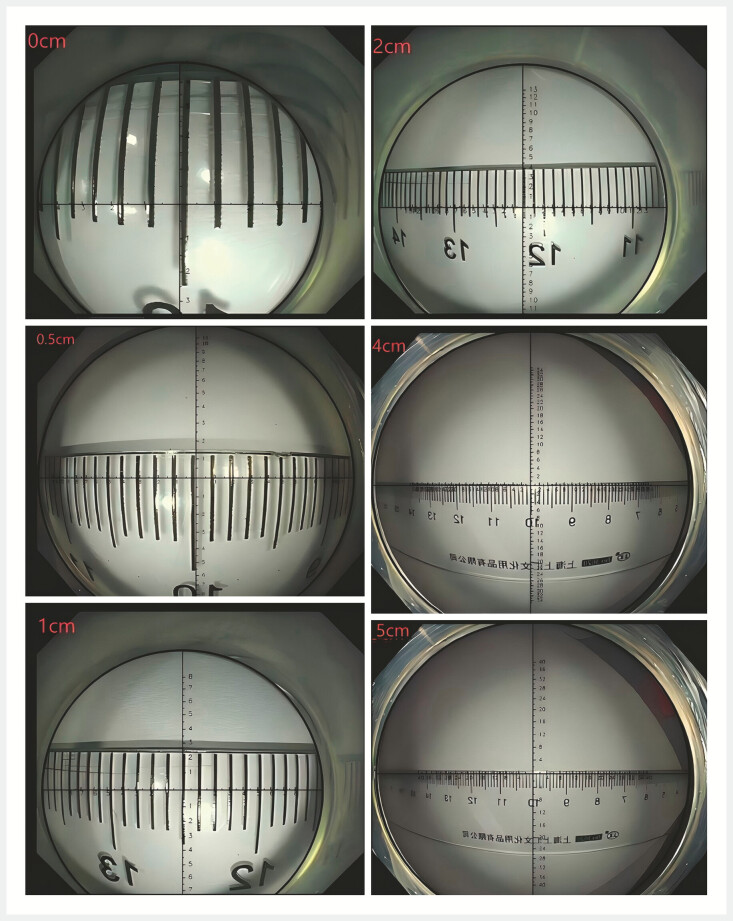
Comparison of the scale of the virtual ruler and that of the physical ruler at different distances.

Endoscopy_UCTN_Code_TTT_1AQ_2AB

## Notes

Endoscopy E-Videos https://eref.thieme.de/e-videos
*E-Videos* is an open access online section of the journal *Endoscopy* , reporting on interesting cases and new techniques in gastroenterological endoscopy. All papers include a high-quality video and are published with a Creative Commons CC-BY license. Endoscopy E-Videos qualify for HINARI discounts and waivers and eligibility is automatically checked during the submission process. We grant 100% waivers to articles whose corresponding authors are based in Group A countries and 50% waivers to those who are based in Group B countries as classified by Research4Life (see: https://www.research4life.org/access/eligibility/ ). This section has its own submission website at https://mc.manuscriptcentral.com/e-videos .
